# Perspectives on pediatric congenital aortic valve stenosis: Extracellular matrix proteins, post translational modifications, and proteomic strategies

**DOI:** 10.3389/fcvm.2022.1024049

**Published:** 2022-11-10

**Authors:** Cassandra L. Clift, Janet Saunders, Richard R. Drake, Peggi M. Angel

**Affiliations:** ^1^Department of Cell and Molecular Pharmacology and Experimental Therapeutics, Medical University of South Carolina, Charleston, SC, United States; ^2^Division of Cardiovascular Medicine, Center for Interdisciplinary Cardiovascular Sciences, Brigham and Women’s Hospital and Harvard Medical School, Boston, MA, United States

**Keywords:** extracellular matrix, post-translational modifications, proteomics, aortic valve, stenosis, pediatric, congenital

## Abstract

In heart valve biology, organization of the extracellular matrix structure is directly correlated to valve function. This is especially true in cases of pediatric congenital aortic valve stenosis (pCAVS), in which extracellular matrix (ECM) dysregulation is a hallmark of the disease, eventually leading to left ventricular hypertrophy and heart failure. Therapeutic strategies are limited, especially in pediatric cases in which mechanical and tissue engineered valve replacements may not be a suitable option. By identifying mechanisms of translational and post-translational dysregulation of ECM in CAVS, potential drug targets can be identified, and better bioengineered solutions can be developed. In this review, we summarize current knowledge regarding ECM proteins and their post translational modifications (PTMs) during aortic valve development and disease and contributing factors to ECM dysregulation in CAVS. Additionally, we aim to draw parallels between other fibrotic disease and contributions to ECM post-translational modifications. Finally, we explore the current treatment options in pediatrics and identify how the field of proteomics has advanced in recent years, highlighting novel characterization methods of ECM and PTMs that may be used to identify potential therapeutic strategies relevant to pCAVS.

## Introduction

Congenital aortic valve stenosis (CAVS) accounts for 10% of all congenital heart defects, which effect 1 in every 150 people (1). The dominant phenotype of CAVS is the fusion of two of the three leaflets, causing a narrowing of the aortic orifice, obstructing aortic outflow, leading to left ventricular hypertrophy and heart failure. Despite the clinical significance of this disease, no pharmacotherapeutics exist. The clinical paradigm for treatment is “watch and wait” until surgical valve replacement and repair is necessary to prevent heart failure. Most CAVS patients are stable until adulthood when they develop fibrocalcific AVS (FAVS), where the end-stage is valve calcification (2). However, ∼10% of all CAVS patients are pediatrics (pCAVS), where end-stage is defined as excessive ECM deposition. Bioengineered AV replacements are notably limited for pediatric patients due to their inability to grow with the patient, a lifelong requirement of anti-clotting agents, and repeated surgeries due to valve failure (3). There remains a critical need to identify pharmacotherapeutic targets in pCAVS that may halt disease progression. One knowledge gap is of collagen regulation in valvular disease. Collagens are the fundamental scaffolding of valvular structure that influences valvular, and thus cardiac function (4, 5). In the future sections, we aim to outline ECM regulation, with a focus on collagen subtypes, in valvular health and disease.

## Healthy aortic valve development and structure

### Structural extracellular matrix in aortic valve development

Just after the initial heart contractions occur (E16–22 days) in early embryonic valve development, the three endocardial aortic cushions form (E31–35 days) and begin to emerge as ECM is deposited. At this time, the aortic valves (AVs) are primarily composed of hydrophilic glycosaminoglycans (GAGs), hyaluronan (HA), lined by endothelial cells (6–10). Between E35 and 37, hyaluronan synthase-2 (Hyal2) activity (which catalyzes formation of the HA polymer) is elevated, forming a “jelly” in which cells of the developing AVs undergo endothelial to mesenchymal transition (EndMT) (6, 10–12). This results in two unique populations of cells – valvular endothelial cells (VECs) and valvular interstitial cells (VICs). As healthy valves are avascular, VECs are responsible for regulating nutrients and biochemical signals from the blood to the VICs (13). VICs organize and maintain valvular structure through regulation of ECM proteins (14–18). Studies show intercellular crosstalk between VECs and VICs is essential to valvular health (19–21). Additionally, cardiac neural crest cells (CNCCs) and second heart field (SHF) precursors migrate to the newly formed endocardial cushions, promoting cell apoptosis and ECM regulation of the developing valve (17, 22–24). Recent literature also shows a population of mesenchymal stem cells present within the human-derived aortic valve cultures (25). At E37–E42, cell populations concurrently proliferate and apoptose, additional ECM is deposited, and the endocardial cushion elongates (5, 26–31).

**FIGURE 1:**
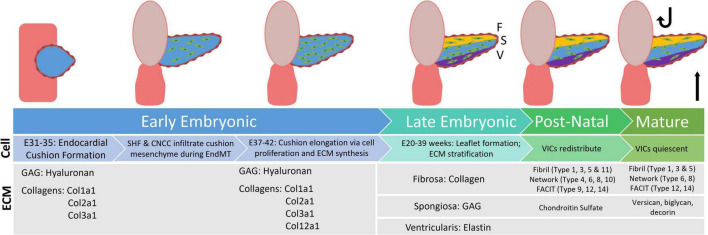
Flowchart displaying distribution of extracellular matrix proteins during the stages of valve development, from early embryonic through the mature aortic valve. GAG, glycosaminoglyca; SHF, second heart field; CNCC, cardiac neural crest cells; EndMT, endothelial to mesenchymal transition; VIC, valvular interstitial cells [development information edited from Combs et al. (6). ECM information edited from Peacock et al. (4)].

In late embryonic AV development (E20–39 weeks for humans) the valve leaflet stratifies into three distinctive layers – the collagen rich fibrosa, the GAG rich spongiosa, and the elastin rich ventricularis. Studies in mice have shown that ECM organization begins during embryonic development and continues after birth, when oxygen levels and hemodynamics (and corresponding biomechanics) adjust (5). Throughout development, the composition and distribution of GAG subtypes change. The once prominent HA is supplemented with chondroitin sulfate proteoglycans, such as aggrecan and versican, as well as small leucine-rich proteoglycans such as biglycan and decorin, amongst others (4, 32). Similarly, collagen type distributions change over the course of development. While fibril-type collagens Col1a1, Col1a2, and Col3a1 are dominant in early development, later embryonic and post-natal development involves the deposition of network and FACIT type collagens as well (Figure 1). This data, however, is interpreted from mouse studies (4). Similar studies have yet to be done extensively on post-natal human aortic valve tissues. In this review we focus on collagen-related changes in heart valve development and disease.

### Role of hemodynamics and gene expression on valve structure

The aortic valve is the most mechanically stressed tissue in the body, undergoing approximately 35 million cyclic openings every year. In the mature valve during systole, total stresses reach up to 50 kPa; these stresses can range from 200 to 400 kPa during diastole (calculated based on human valve dynamics) (14, 33). During late embryonic development and post-natal development, peak shear stresses range from 30 to 1,500 dynes/cm^2^ (with higher values occurring during stenosis); these hemodynamics play a significant role in valve structure and stratification (14, 34). During systole, shear stresses are unidirectional and pulsatile, stretching the leaflet, which primarily affects the elastin rich ventricularis. Conversely, the outflow side of the aortic valve requires the stiffer collagen rich fibrosa to maintain structure and function, as oscillatory vortexes develop behind the leaflet during diastole. The internal middle layer of the valve is a GAG rich spongiosa that acts as shock absorbers during the entire cardiac cycle (35). Hemodynamics play a role in collagen alignment in the healthy valve, which has been shown to align along the circumferential axis, parallel to the endothelium (36, 37). In addition to hemodynamics, emerging subpopulations of VICs and differentially expressed genes can play a role in ECM stratification. In the ventricularis, Notch1 expression is dominant and may play a role in elastin deposition (6, 38). In the spongiosa layer, BMP2 signaling promotes Sox9 expression and deposition of proteoglycans, such as aggrecan, versican, chondroitin sulfate, and decorin (6, 39). In the fibrosa layer, Wnt signaling has been linked to promote the expression of fibroblast related ECM components such as fibrillar collagens type 1 and 3 and fibronectin (6, 40).

### Structural regulation of post-translational modifications on extracellular matrix

**FIGURE 2:**
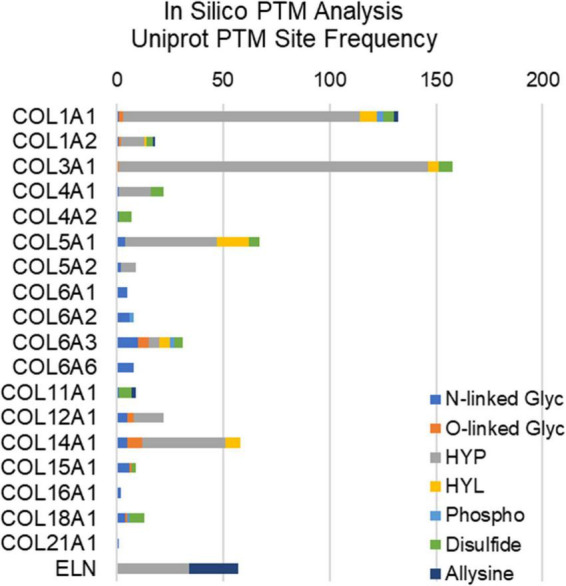
*In silico* post-translational modification (PTM) site frequency analysis of valvular structural proteins (collagens and elastin); uniport reporting. Glyc, glycosylation; HYP, hydroxyproline; HYL, hydroxylysine; Phospho, phosphorylation.

Extracellular matrix proteins make up the bulk of valvular tissues, and these proteins are abundantly post-translationally modified (Figure 2). Hydroxylation is a major post-translational modification (PTM) of collagens that maintains structural integrity of the valve. Hydroxylation of proline and hydroxylation of lysine are the two most abundant PTMs of collagen type proteins (41, 42). These PTMs are necessary for the organization of collagen’s triple helix structure as well as its thermal stability. In both collagen and elastin, hydroxyproline is a well-regulated modification, with between 20 and 24% of prolines being hydroxylated (41). Prolyl hydroxylases are responsible for hydroxylation of proline in elastin as well as collagen, with an X–P–G amino acid sequence required substrate in most cases (43). Elastin studies of the human aorta show that two specific prolines (P190, P615) have the highest degrees of hydroxylation, suggesting site-specific regulation, however, this has not been elucidated in the healthy or diseased AV (44). Similarly, only a single study has attempted to perform site specific hydroxyproline mapping in health or diseased AV (37). Other PTMs of structural ECM in the valve include *O*- and *N*-linked modifications, phosphorylation, allysine via lysyl oxidase, and collagen-lysyl intermolecular crosslinking (Figure 2). This other ECM PTMs have been attributed to site-specific functions related to protein folding, ECM secretion in stress conditions, collagen assembly, crosslinking and stabilization.

This review primarily focuses on major structural ECM proteins (collagen and elastin). For information regarding minor ECM proteins, glycoproteins, and proteoglycans (such as vitronectin, fibronection, periostin, osteonectin, osteopontin, and aggrecan) basement membrane proteins (laminin, perlecan, and nidogen) or ECM remodeling proteins, such as matrix metalloproteinases (MMPs) and their inhibitors (TIMPs), we point the reader to the following reviews and original research manuscripts (9, 36, 45–52).

## Congenital aortic valve stenosis

Currently, congenital aortic valve stenosis (CAVS) accounts for 10% of all congenital heart defect cases, which affect 1 in 150 people (1). CAVS progresses as an obstructive narrowing of the aortic opening due to enlargement of the AV leaflets through deregulated ECM production and results in heart failure. Approximately 90% of all CAVS cases are due to bicuspid aortic valve (BAV) (53). Disease loci associated with BAV have been found on chromosomes 18q, 13q, and 5q and while the inheritability of BAV within certain families suggest a genetic component, only mutations in NOTCH1 signaling have been attributed directly to valvular stenosis (54, 55). However, other genetic mutations have been associated with BAV-aortopathy (56). Despite other genes being implicated in murine models of BAV (such as eNOS and GATA6 mutations), these have not been correlated to the valvular stenosis in humans (57, 58).

### Pediatric versus adult congenital aortic valve stenosis

**FIGURE 3:**
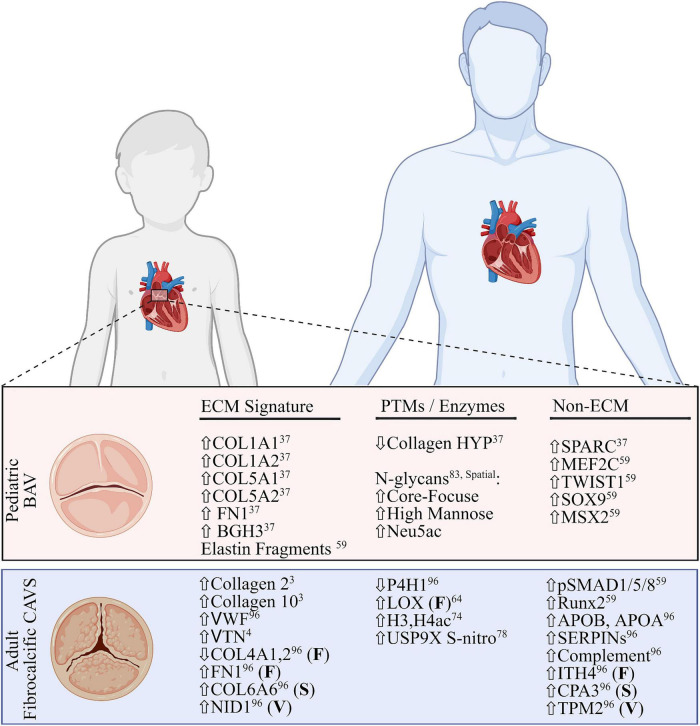
Comparative summary of transcriptomic and proteomic profiles of pediatric bicuspid aortic valve disease and adult fibrocalcific CAVS. Extracellular matrix (ECM) signatures **(left)**, post-translational modifications or related enzymes **(middle)** and non-ECM/PTM signatures **(right)** are shown. All signatures are described as global changes unless otherwise specified (F, fibrosa; S, spongiosa; V, ventricularis). For the purposes of this review, the top layer-specific alteration is shown. FN1, fibronectin; BGH3, TGFB induced protein ig-h3; MEF2C, myocycte-specific enhancer factor 2C; TWIST1, twist-related protein 1; MSX2, homobox protein MSX2. VWF, Von Willebrand Factor; VTN, vitronectin; NID1, nidogen-1; P4H1, prolyl-4 hydroxylase-1; LOX, lipoxygenase; H3,H4, histone3,4; USP9X, ubiquitin carboxyl-terminal hydrolase FAF-X; Runx2, runt-related transcription factor-2; ITH4, inter-alpha trypsin inhibitor heavy chain 4; CPA3, carboxypeptidase A3; TPM2, tropomyosin 2. Figure created using BioRender.com (2022).

Most CAVS patients (90%) are stable until adulthood, where severe calcific lesions are the primary cause of valve replacement. In adult fibrocalcific AVS (FAVS), inflammation and adhesion are shown to increase along with mast cell infiltration (59, 60). Adult cases also tend to have comorbidities, such as high blood pressure or coronary artery disease, further confounding research (54). The remaining 10% of all CAVS patients are pediatrics, where end-stage is defined by excessive ECM deposition, and no calcification (pCAVS) (59, 61). A main feature of both pediatric and adult CAVS is bicuspid aortic valve (BAV). The dominant BAV morphology across all patients is the fusion of the right- and left-coronary leaflets (71%) (62). Potential changes in dominant cusp fusion morphology (RL, RN, and NL) have not been differentiated between pediatric and adult calcific CAVS progression; however, right and non-coronary fusion (R–N) are the most common predictor of pediatric valve dysfunction and intervention (35% of patients; >4-fold increase) (63). Literature acknowledges a distinct molecular profile between pediatric and adult calcific CAVS (59), but the molecular mechanisms behind the rapid progression of pCAVS remain to be elucidated. A summary of what is known regarding distinct molecular signatures between these two groups is outlined in Figure 3.

### Congenital aortic valve stenosis: Changes to valvular extracellular matrix architecture

A loss of ECM stratification is seen in both pediatric and adult cases of CAVS (59). Hutson et al. set out to quantify changes in collagen architecture associated with adult calcific cases of CAVS (64). Their group showed that collagen content in the GAG-dominant spongiosa layer doubled in the disease state. Collagen fiber width and density also significantly increased, with lysyl oxidase expression also increasing in the diseased fibrosa layer (64); however, the corresponding PTM allysine was not studied. Studies in mice have shown that loss of crosslinking collagens Col5a1 and Col11a1 alter expression of fibrillar collagens type 1 and 3, resulting in AV stenosis (54). Similarly, studies of elastin in CAVS has shown increased fragmentation correlated to increased calcification (65). Fragmented elastin has also been seen in pediatric CAVS (59). Di Vito et al. recently published an extensive review of ECM alterations in adult calcific valve disease (66). Our group recently reported that total collagen deposition increases in pediatric CAVS, along with collagen fiber misalignment relative to the endothelium, and discrete regions of increased collagen fiber density (or “plaques”), as measured by histology (37). ECM targeted proteomics in this study showed differential collagen subtype regulation (in COL1A1, COL1A2, COL5A1, and COL5A2) between pCAVS, normal, and tricuspid aortic valve insufficiency (37). The role of site-specific collagen PTMs has yet to be studied in a localized manner relative to these collagen plaques, or layer specificity.

### Altered hemodynamics in bicuspid aortic valve contribute to deregulated expression

Congenital aortic valve stenosis-mediated alteration in hemodynamics influences all levels of molecular expression and feeds forward into deregulating the valvular structure (62). Velocity field analysis has shown that the total kinetic energy, Reynolds shear stress, and viscous shear stress are all significantly higher in cases of BAV as compared to the normal tricuspid configuration (35). *Ex vivo* analysis shows that abnormal hemodynamic shear stress may contribute to early adults CAVS pathogenesis (67). Additionally, pediatric and adult cases of BAV experience similar hemodynamic alterations (68). In environments with excessive shear stress such as the AV, enzyme-induced turnover of ECM is essential to maintain homeostasis. However, shear stresses experienced in BAV have been shown to exhibit myofibroblast induced-remodeling, attributed excessive ECM deposition (69). There remains a need to explore how similar shear stresses experienced in BAV effect ECM expression during different developmental stages, recapitulating pediatric vs. adult BAV fluid responses.

During *in vitro* bioreactor conditioning studies, increased pressure has been directly attributed to increased leaflet strain (70). Studies in VICs under variable strain (10, 14, and 20%) show significant increase in [^3^H]-proline incorporation, indicating increased collagen synthesis (70), but hydroxyproline site specificity in bioreactors conditions has yet to be explored. Transcriptional regulation of critical genes differs during strain as well. For example, Ku et al. showed Col3a1 is significantly upregulated as compared to a steady regulation of Col1a1 under strain (71). These studies offer insight into how the increased shear stresses seen in BAV contribute altered strain and to ECM deposition in CAVS.

### Role of post translational modifications in cardiac development and disease

The role of PTMs in ECM proteins is beginning to be explored in valve development and disease. While the PTM allysine has not been measured directly in CAVS, studies show an increase in the enzyme responsible for catalyzing the reactive aldehyde species, LOX (64). Similarly, PLOD1, the enzyme responsible for catalyze hydroxylysine residues in collagen, was found to be unchanged in adult CAVS compared to controls (64), however, hydroxylysine was not measured directly. While hydroxyproline content has been measured in healthy aortic valves, this is used as a measurement of insoluble collagen content, not in a site-specific analysis (72, 73). Our group recently reported the first study of collagen HYP quantification and site mapping with human pediatric CAVS tissue (37). Overall, a reduction of collagen HYP content was seen, particularly in regions within collagen sequence required for integrin and glycoprotein vi binding (37).

Potential modifications of intracellular proteins regulated in the heart can include both enzymatic (such as acetylation, phosphorylation, and ubiquitination) and non-enzymatic (such as nitrosylation) post-translational modifications. Acetylation of histones 3 and 4 has been reported in CAVS and non-calcified AVs from patients, although significantly higher in CAVS patients (74). Similarly, acetylation plays a role in modulating the heart’s response to I/R injury (75). Glycogen Synthase Kinase 3β (GSK3β) – which has been found in cardiac fibroblast and implicated in ventricular remodeling – is both phosphorylated and *S*-nitrosylated in a site-specific manner relevant to its function in cardiac fibrosis (76, 77). *S*-nitrosylation of USP9X has been shown to be enriched in NOTCH regulation of valvular calcification (78). Additionally, studies have shown increases in ubiquitin-mediated proteolysis in heart failure (79). While these studies are not specific to ECM proteins, data suggested that PTMs may be a potential therapeutic target in cardiac and valvular fibrosis.

### Oxidative stress in valvular disease and potential effect on extracellular matrix proteins and post translational modifications

Post-translational regulation via oxidation has been pinpointed as having a major role in other fibrotic diseases (80). While inflammatory-derived Reactive Oxygen Species (ROS) has been implicated in adult CAVS, ROS is unexplored in pediatric valve disease (66). However, ECM and PTM studies in pediatric valve disease suggest a potential role for ROS regulation. ROS can alter the expression of ECM both by activating transcription factors involved in ECM protein expression (such as TGFB1) as well as modifying ECM proteins post-translationally (80). Additionally, the hydroxylation of proline and lysine residues of collagen is highly redox dependent. Once lysine is hydroxylated, it serves as a synthesis starting point for proteins responsible for collagen and elastin crosslinking, such as desmosine (42, 80, 81). Hydroxylated lysine 2 has been shown to have glycosyl-transferase activity, serving as an anchor for *O*-glycosylation modification (41, 42). *In silico* analysis of valvular collagen type proteins demonstrates a potential abundance of *N*- and *O*-glycosylation sites, amongst other PTMs (Figure 2). At high concentrations, ROS can destroy these carbohydrate entities (80, 82). A recent publication by Angel et al. shows that *N*-glycosylation patterns are spatial regulated in pediatric aortic valve development and disease, with certain sialylated *N*-glycans amongst others being upregulated (83). At low concentrations, ROS can induce GAG-degrading enzymes, such as Hyal2, indirectly effecting ECM turnover (32). Recent studies have shown that GAGs can store growth factors and cytokines in the extracellular space; so, indirectly, depletion of GAGs via Hyal2 may modify receptor binding and activation (84). ROS can also induce collagen degrading enzymes, such as matrix-metalloproteinase (MMPs), further suggesting a role of oxidative stress in ECM remodeling (85).

## Current treatment options and bioengineered solutions

### Valve replacement options for pediatric congenital aortic valve stenosis

**FIGURE 4:**
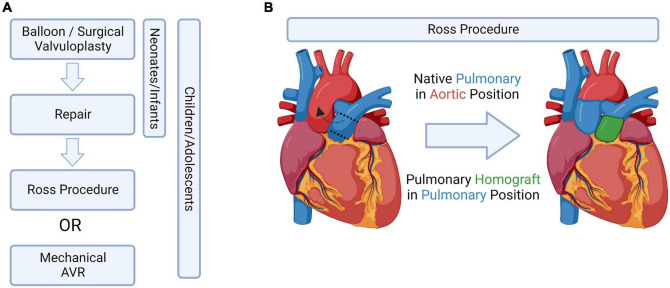
Current treatment options, bioengineered solutions, and the Ross Procedure. **(A)** Potential aortic valve intervention strategies in pediatric patients, adapted from Bouhout et al. (91). **(B)** Schematic drawing of the Ross procedure, which switches the native pulmonary valve into the aortic position. Figure created using BioRender.com (2022).

Despite the clinical significance of CAVS, surgical valve replacement and repair is still the only available treatment. Mechanical-based engineered valve replacement devices are not suitable for young children as they lack any growth potential as well as requiring lifelong anti-coagulation therapy (86). However, in older pediatric cases, there has been success with a 55–90% freedom from reoperation at 15 years (3). Tissue engineered valve replacements are currently unavailable in sizes smaller than 19 mm, also making them unsuitable for young pediatric cases. Like mechanical prosthesis, there is a lack of growth potential with tissue engineered replacements, but anti-coagulation therapy is not required (87). Survival was reported to be 85% at 10 years, however the rate of reoperation is high – 65% at 10 years, with the median longevity being only 7.5 years (3). Homografts are able to retain normal hemodynamic profiles over time, with small sizes available for small children. Homografts also do not require anti-coagulation therapy and have the added advantage of being more resilient to infection that tissue engineered prosthesis, however, there is a limited donor pool (3, 88). Despite these advantages, homografts show only a 60% survival at 10 years, and 30% at 20 years and are not ideal for pediatric cases (89). Current therapeutic strategies are highlighted in Figure 4.

### The Ross procedure and mechanisms of failure

The limitations listed above are why the Ross procedure, which switches the native pulmonary valve into the aortic valve position, is currently the preferred treatment amongst young pediatric cases (90–92) (Figure 4B). However, this procedure does not come without complications: children having undergone this procedure have a 14.3% mortality rate in the first year – after surviving the first year, the likelihood of the child needing an aortic or pulmonary reintervention later in life is 15.3 and 27.5%, respectively (3).

Mechanisms of failure of pulmonary valves in the aortic position during Ross procedures can be inferred from studies regarding proteomic differences between the two valves. Ikhumetse et al. found that when pulmonary valve derived VICs were exposed to cyclic aortic pressures, sulfated GAGs as well as collagen synthesis were significantly increased (93). Angel et al. aimed to characterize the difference between AV and PV via chromatographic proteomic approaches. The study found that only 50% of the proteome of the AV is shared with the PV. The PV proteome was abundant in hypoxia associated proteins such as H2AX but only sparingly expressed proteins that may be critical to healthy AV function, such as MFGe8 and Emilin-1 (94). The role of PTMs in surgical valve failure has not been explored.

## Future directions: Proteomic characterization and identification

**FIGURE 5:**
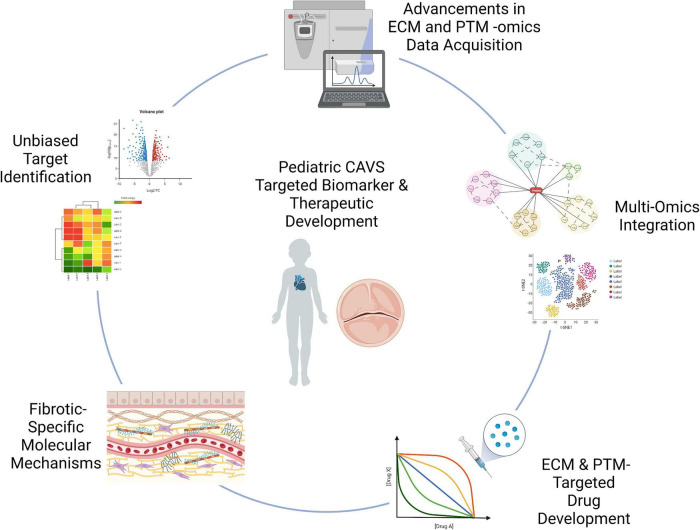
Graphical summary of technological advancements to be applied to pCAVS. Unbiased approaches, such as proteomics and transcriptomics, allow for novel discovery of disease markers. Advancements in acquisition techniques as described allow for a deeper mining of the extracellular matrisome, previously limited in accessibility. This increased accessibility allows for identification of post-translational modifications; while multi-omics integration probe upstream regulating enzymes, accelerating identification of therapeutic targets. Extracellular matrix targeted -omics techniques have the potential to elucidate fibrosis-specific biomarkers, highlighting the distinct molecular mechanisms between adult calcific and pediatric fibrotic CAVS. Graphics created using BioRender.com (2022).

While transcriptional regulation of the valvular ECM has been well characterized, low cell density and high ECM content warrant translational level studies. Translational and post-translational regulation of these proteins in pediatric CAVS remains to be fully elucidated, as the majority of literature focuses on adult calcific cases of CAVS. Acquiring information on spatial organization of molecular patterns of ECM proteins and their PTMs is essential to defining both disease and development. In this section we aim to review advancements in the field of valvular and extracellular matrix focused proteomics, the relevance of spatial -omics in valve disease, and how multi-omics analysis may help identify novel therapeutic targets relevant to pediatric valve fibrosis (Figure 5).

### Proteomic characterization of extracellular matrix proteins in valve tissues and serum

The ECM proteome in pediatric valvular tissue has been difficult to characterize via conventional tryptic methods due to high levels of intra- and intermolecular crosslinking and PTMs. Brioschi et al. published a comparative analysis of gel-based and gel-free proteomic methods to characterize the human mitral valve proteome, showing large variability between the four methods and only 12% of all proteins identified were associated with ECM pathways (95). Schlotter et al. used label-free tryptic proteomics techniques with isolated VICs and micro-dissected valvular tissues. Amongst other findings, they showed that collagen biosynthesis, collagen modification, GAG metabolism, and ECM degradation pathways were enriched in the adult CAVS isolates (96). While these methods can implicate ECM pathways, ECM proteins were not specifically enriched during sample preparation for these studies. After years of advancement in valvular proteomic techniques (97, 98), the Barderas Lab enriched for ECM proteins in their 2D-LC MS/MS method on calcific AVS samples. The group found 13 differentially regulated ECM proteins in the adult calcific CAVS samples compared to controls. Of these, biglycan, periostin, prolargin, decorin, and lumican (implicated in collagen fiber assembly) formed a protein–protein interaction network cluster. One collagen protein, Col6a1, was identified to be upregulated in adult CAVS (99). Similarly, Bouchareb et al. used guanidine hydrochloride to extract the ECM for proteomic analysis, and identified two potential biomarkers of adult calcific CAVS – FNDC1 and MXRA5 (100). While not on native valvular tissue, Syedain et al. recently performed ECM enriched proteomic analysis on tri-tube valve conduits to analyze their regenerative capacity (101). The Hansen Lab recently did a comparative study of a dozen ECM enrichment techniques for proteomics, comparing decellularization, extraction and single-shot analysis methods (102). Alternative enrichment approaches used by our group use ECM-targeting enzymes, such as collagenase type III and MMP12, to enzymatically enrich for the extracellular matrix within valvular tissues for both spatial and LC-MS/MS proteomics. These studies have been applied to clinically available pediatric BAV formalin-fixed paraffin embedded (FFPE) tissues, with all proteins identified being of the extracellular matrix, and roughly half being collagen subtypes (37).

Serum proteomics is a potential source for biomarker discovery in tissue diseases unable to be biopsied, such as valvular disease. To date, no proteomic analysis has been done on serum correlating to pediatric CAVS tissue. However, serum profiling has been done for cardiac failure (103, 104) and other fibrotic diseases such as idiopathic pulmonary fibrosis (105) and liver cirrhosis (106). While there are many large-scale proteomic studies of serum profiles, ECM proteins are inherently difficult to enrich for (107). Multimodal -omics approaches, such as epigenomics, transcriptomics, proteomics, and metabolomics, offer promising advantages to traditional characterization techniques, and together moves the field away from strictly characterization toward biomarker and pharmacotherapeutic target discovery, as well as to inform bioengineered solutions (108, 109).

### Spatial localization of extracellular matrix proteome: Linking structure to function

Despite advancements in multi-omics techniques listed above, bulk LC-MS/MS proteomics still exclude a key variable of disease pathology – spatial localization of dysregulated ECM within the tissue microarchitecture. Several mass spectrometry techniques exist that link the tissue proteome to its structure. One technique is Matrix-Assisted Laser Desorption/Ionization (MALDI) Imaging Mass Spectrometry (IMS). MALDI-IMS is a robust proteomic technique that spatially maps peptides from histological tissue sections. This is traditionally done by spraying the tissue with a thin, uniform layer of enzyme that, when analyzed in parallel to high resolution accurate mass proteomics, allows for the relative quantitation and localization of peptides and other analytes to specific regions of the tissue. In addition to tryptic peptides, novel MALDI-IMS and LC-MS/MS techniques have been developed to specifically map ECM proteins, that until recently were unable to be accessed via conventional proteomic workflows (37, 110–115).

Proteomic imaging via MALDI-IMS may allow for greater protein identification and improved PTM evaluation compared to antibody-based histology strategies. Angel et al. recently outlined the challenges of analyzing cardiac tissues with MALDI-IMS as well as the advancements that have been made in the field (116). Mourino-Alvarez et al. recently took advantage of MALDI-IMS technology to characterize fresh frozen calcific AVS tissue. The group was able to visualize m/z peaks correlating to calcified, collagenous, and elastin rich tissue areas. Two peptides of interest were from Col6a3 (responsible for calcium and hydroxyproline accumulation on human osteoblast-like cells) and NDRG2 (implicated in p53 mediated apoptosis and mineralization initiation) (117). Recently, several studies have emerged using MALDI-IMS and other imaging mass spectrometry methods to characterize molecules associated with healthy and diseased aortic valve, such as *N*-linked glycans (83, 112), lipids (118–120), glycosaminoglycans (112), and peptides (110, 112, 116). With diseased valve tissue samples becoming less abundant on the bench due to increases in transcatheter aortic valve replacements (121), these multi-modal MALDI-IMS techniques are a promising tool that can be used to characterize ECM deposition and post-translational modifications during CAVS progression, in conjunction to histopathological evaluation (111, 112).

### Emerging role of collagen post-translational modifications in disease

Post translational modifications of ECM have been linked to various disease states, not limited to fibrotic diseases. While hydroxyproline and hydroxylysine modifications are essential to proper collagen fibril formation, over-hydroxylation of the protocollagen chains can delay collagen polymerization, resulting in excessive PTMs, such as glycosylation (41, 42, 122). This perturbs collagen’s triple-helical structure further which in turn contributes to various disease states, such as Ehlers-Danlos syndrome and osteogenesis imperfecta (41, 123). Of note, collagen-dysregulation associated connective tissue disorders have a high prevalence of aortic valve defects, including aortic valve stenosis (124). Similarly, under-hydroxylation can destabilize collagen’s triple helix, also contributing to disease states, as seen in vitamin C deficiencies leading to scurvy (125). Hydroxyproline modifications in collagen amongst other proteins has been implicated in oxygen-sensing pathways in cancer cells (126). Hydroxylysine aldehyde–derived collagen cross-links have been found to be upregulated in lung tumor stroma (127). Kamel et al. shows that increased hydroxylation of lysine in collagen increases the proliferation rate of uterine fibroids (128). Additionally, increased tissue stiffness due to over hydroxylation of the ECM may not only contribute to progression of tumors and fibrotic diseases but may also influence diffusion of drug molecules within the disease microenvironment (129, 130). Very few approaches are able to map which sites of hydroxylation contribute to disease status when modified. For these reasons, characterization of not only the ECM, but also site-specific PTMs, are critical for elucidating mechanisms of fibrotic diseases, such as pediatric end-stage CAVS.

### Targeting extracellular matrix proteins and post translational modifications as a therapeutic strategy

Identified therapeutic strategies for adult CAVS, such as targeting of circulating lipid levels or osteogenic differentiation of VICs and mineral deposition directly, would not be appropriate for targeting the fibrosis seen pediatric end-stage (131). The late diagnosis of fibrotic diseases limits therapeutic intervention before rapid ECM remodeling occurs. The future of fibrotic treatments may rely on proteomic strategies to identify new ECM biomarkers, resulting in earlier detection. Walraven and Hinz propose in their 2018 review that inhibiting fibrosis will depend on two main strategies: (1) targeting the myofibroblasts directly through stimulatory or suppressing cytokines and stimulating apoptosis; and/or (2) targeting the fibrotic ECM directly using targeted proteolytic enzymes (132). Currently, the only FDA approved drugs on the market to treat any fibrotic disease (idiopathic pulmonary fibrosis) are Nintedanib and Pirfenidone. Previously thought to work through kinase-dependent fibrotic pathways (133), a recent study shows that these drugs inhibit collagen fibril assembly directly (134). A recent study suggests that clinically targeting the denatured collagen, which occurs in fibrotic disease during enzymatic remodeling, may be possible via collagen mimetic peptides or antibodies (135). Another proposed anti-fibrotic therapeutic strategy is the targeting of hydroxyprolines via collagen prolyl-4-hydroxylase (CP4H). However, CP4H inhibitors lack selectivity – while promising results are seen *in vivo*, off target affects to redox homeostasis and iron metabolism are also seen (136). Other strategies to target hydroxyproline involve scavenging the ROS superoxide, which inhibits CP4H (137, 138). Alternatively, targeting collagen-related partners whose binding is dependent on hydroxyprolination, such as integrins or glycoproteins, may modulate fibrotic signaling (138–140). While the role of hydroxylysine and allysine have yet to be elucidated in pCAVS or fibrocalcific CAVS, inhibition of lysyl oxidase inhibitors may be an additional potential therapeutic strategy, with promising results shown related to fibroblast activation in liver fibrosis (138). Realistically, the goal of anti-fibrotic therapies in pediatric CAVS may not be to cure stenosis but instead to delay the severity until patients reach an appropriate age and size for bioengineered options to be used without the need for repeated surgeries.

## Conclusion

A major gap in knowledge hindering the development of therapies for pediatric CAVS is that the molecular mechanisms underlying the structure-function relationship remain poorly defined. One therapeutic avenue in need of further scientific exploration is the targeting of extracellular matrix proteins and their post translational modifications – specifically, collagens. Collagens are the fundamental scaffolding of valvular structure that influences valvular function, and thus cardiac function (4, 5). Multiple collagens are known for their cardiac remodeling potential and are involved in valvular development (7, 141, 142), yet translational regulation of these critical proteins due to pediatric CAVS remains unknown – thus preventing appropriate drug targets from being developed and administered upon initial diagnosis. Advancement of high-resolution accurate mass proteomics and spatial techniques like MALDI imaging mass spectrometry offer unique advantages in understanding the structure/function relationship of the ECM in cardiac tissue. These novel strategies may provide insight into AV development, the rapid progression of pediatric end-stage CAVS.

## Author contributions

CC and PA: conceptualization and writing. CC, JS, RD, and PA: review and editing. RD and PA: supervision. CC, RD, and PA: funding acquisition. PA: project administration. All authors have read and agreed to the published version of the manuscript.
